# Small intestinal microbiota composition altered in obesity-T2DM mice with high salt fed

**DOI:** 10.1038/s41598-023-33909-2

**Published:** 2023-05-22

**Authors:** Goher Kerem, Xiangfang Yu, Aynur Ismayi, Bin Teng, Anjaneyulu Udduttula, Chang Liu, Zhongjia Yu, Dilbar Tohty, Jian V. Zhang, Pei-Gen Ren

**Affiliations:** 1 Xinjiang Key Laboratory of Special Species Conservation and Regulatory Biology, College of Life Science, Xinjiang Normal University, Urumqi, China; 2 Center for Energy Metabolism and Reproduction, Shenzhen Institutes of Advanced Technology, Chinese Academy of Sciences, Shenzhen, China; 3 Shenzhen College of Advanced Technology, University of Chinese Academy of Sciences, Shenzhen, China; 4 Guangdong Key Laboratory of Nanomedicine, Shenzhen, China

**Keywords:** Microbiology, Diseases

## Abstract

Obesity has become a global concern because of increasing the risk of many diseases. Alterations in human gut microbiota have been proven to be associated with obesity, yet the mechanism of how the microbiota are altered by high salt diet (HSD) remains obscure. In this study, the changes of Small Intestinal Microbiota (SIM) in obesity-T2DM mice were investigated. High-throughput sequencing was applied for the jejunum microbiota analysis. Results revealed that high salt intake (HS) could suppress the body weight (B.W.) in some extent. In addition, significant T2DM pathological features were revealed in high salt-high fat diet (HS-HFD) group, despite of relatively lower food intake. High-throughput sequencing analysis indicated that the *F/B* ratio in HS intake groups increased significantly (*P* < 0.001), whereas beneficial bacteria, such as lactic acid or short chain fatty acid producing bacteria, were significantly decreased in HS-HFD group (*P* < 0.01 or *P* < 0.05). Furthermore, *Halorubrum luteum* were observed in small intestine for the first time. Above results preliminary suggested that in obesity-T2DM mice, high dietary salt could aggravate the imbalance of composition of SIM to unhealthy direction.

## Introduction

Obesity is a condition in which excess or abnormal fat accumulation may present with adverse effects on health, and decreased life expectancy. Increased body weight and adipose tissue accumulation amplifies the risk of various age-related syndromes, such as cardiovascular disease, type 2 diabetes mellitus, musculoskeletal disorders, respiratory illnesses, and certain types of cancer.

Unhealthy eating habits, including high fat, high carbohydrates and high salt diets, have been proven to be major causes for obesity^1^. For instance, long-term high-fat diets (HFD) were the main causes for obesity, and often accompanied with a range of metabolic disorders, such as type 2 diabetes (T2D) and cardiovascular diseases (CVD). It had been well established that chronic high salt diets (HSD) amplified the risk of CVD, blood pressure elevation, and higher morbidity and mortality^2,3^. In addition, studies based on fecal samples had demonstrated that HSD might modify the composition of gut microbiota, and increased the ratio of *Firmicutes*/*Bacteroidetes* (*F/B*), which is one of the biomarkers for pathological condition of CVDs and obesity^4–7^.

The human distal gut harbors a vast ensemble of microbes (the microbiota) that provide important metabolic capabilities, including the ability to extract energy from other indigestible dietary polysaccharides. Gut microbial coexist with human body as mutualists, and changes on gut microbiota composition could cause the alteration of human health^4,8^. The small intestine (SI) is responsible for approximately 90% of the overall energy absorption from the diet. The adequate absorptive capacity of SI depends on maintenance of mucosal integrity that is constantly challenged by its exposure to the luminal milieu, including the microbiota. Most of the previous experiments on identifying microbiota aberrations have been targeting the large intestine and fecal microbiota. Nevertheless, the small intestine microbiota (SIM) might also have a profound impact on various aspects of the host’s physiology, including immune, metabolic and endocrine functions. Moreover, studies have demonstrated the importance of small intestinal microorganisms (SIM), either as transducers of dietary signals, or inducing the alterations of the bacterial population in small bowel and intestinal abnormalities in mouse or rat^9–11^. Previous investigations illustrated a correlation between SIM balance with diseases, dietary nutrition uptake, and function regulations^12,13^. However, the relationship between HSD and SIM alteration in obesity individuals remains to be unclear^14,15^.

In this study, a high salt-high fat diet feeding mouse model and a high-throughput sequencing technology were conducted to investigate the changes of microbial flora in the proximal small intestine of the mice. Moreover, the connection between high dietary salt, SIM, and metabolic syndrome were tentatively discussed based on obtained results.

## Results

### Body weight (B.W.), food and water intake

**Figure 1:**
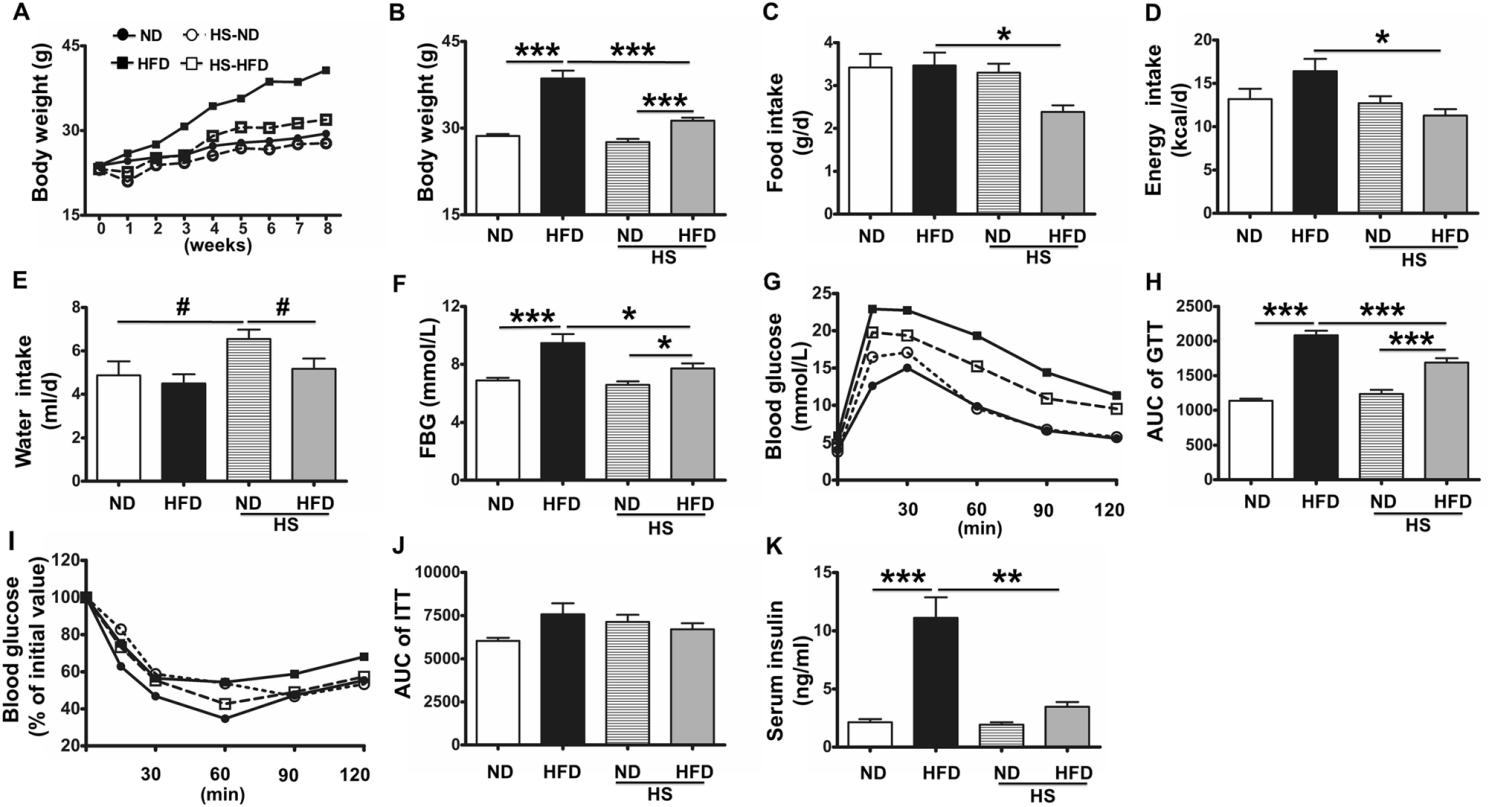
Effects of different diets on mice groups after 8-week feeding. (**A**,**B**) Curve and bar graphs of B.W. of mice feeding with different diet for 8 weeks. (**C**) Food intake. (**D**) Energy intake. (**E**) Water intake. (**F**) Fasting blood glucose (FBG). (**G**,**H**) Curve and bar graphs of glucose tolerance (GTT); bar showed area under the curve (AUC) information. (**I**,**J**) Curve and AUC bar graphs of insulin tolerance (ITT). (**K**) Serum insulin level. ND (n = 10), HFD (n = 9), HS-ND (n = 10), HS-HFD (n = 10). *, ^#^*P* < 0.05, **P < 0.01, ***P < 0.001. Panel (**E**) was measured by Student’s *t* test followed by unpaired *t* test. Other statistics results were measured by one-way ANOVA followed by Tukey’s multiple comparison test.

Before feeding experimental diets, average B.W. of experimental animals were 23 ± 1.75 g. After 8 weeks feeding, the B.W. of four different groups were remarkably different. The most obvious increases were observed in HFD group from 23 to 44 g in 8 weeks (2.63 g/week). The B.W of HS-HFD group was increased from 23 to 34 g (1.76 g/week). Moreover, the B.W of HS-ND and ND groups raised slightly from 23 g to about 28 g (Fig. 1A). Significant statistical differences were revealed between both HFD and HS-HFD groups and their paired ND groups (*P* < 0.001), while no remarkable statistical differences were appeared between HSD-ND and ND groups (*P* = 0.62, Fig. 1B).

For food intake, significantly less food intake was observed in HS-HFD group than HFD group (*P* < 0.05), while a same dietary intake was examined in HSD-ND and ND groups (Fig. 1C). Accordingly, the energy intake for HS-HFD group was less than HFD group (*P* < 0.05, Fig. 1D). In addition, HS-ND group has relatively higher water intake than paired ND and HF-HSD groups (*P* < 0.05), and almost same amount of water intakes were measured in HFD and HS-HFD groups (Fig. 1E).

### Fasting blood glucose (FBG), glucose tolerance (GTT), insulin tolerance (ITT), and serum insulin level

Comparatively higher FBG levels were investigated in both HFD and HS-HFD groups than their correspondent ND groups (*P* < 0.001, and *P* < 0.05), and HFD samples had a higher FBG than HSD group (*P* < 0.05, Fig. 1F). Glucose tolerance tests (GTT) showed that GTT in both HFD and HS-HFD groups were evidently higher than that of two ND groups (*P* < 0.001), and the GTT of HFD group was relatively higher than that of HS-HFD group (*P* < 0.001, Fig. 1G,H). For insulin tolerance, differences were not obvious among the groups (Fig. 1I,J), however, the serum insulin concentrations were significantly increased in HFD group (*P* < 0.001, or *P* < 0.01, Fig. 1K).

### Triglyceride (TG), high density lipoprotein cholesterol (HDL-C), low-density lipoprotein cholesterol (LDL-C) concentration in liver tissue

**Figure 2:**
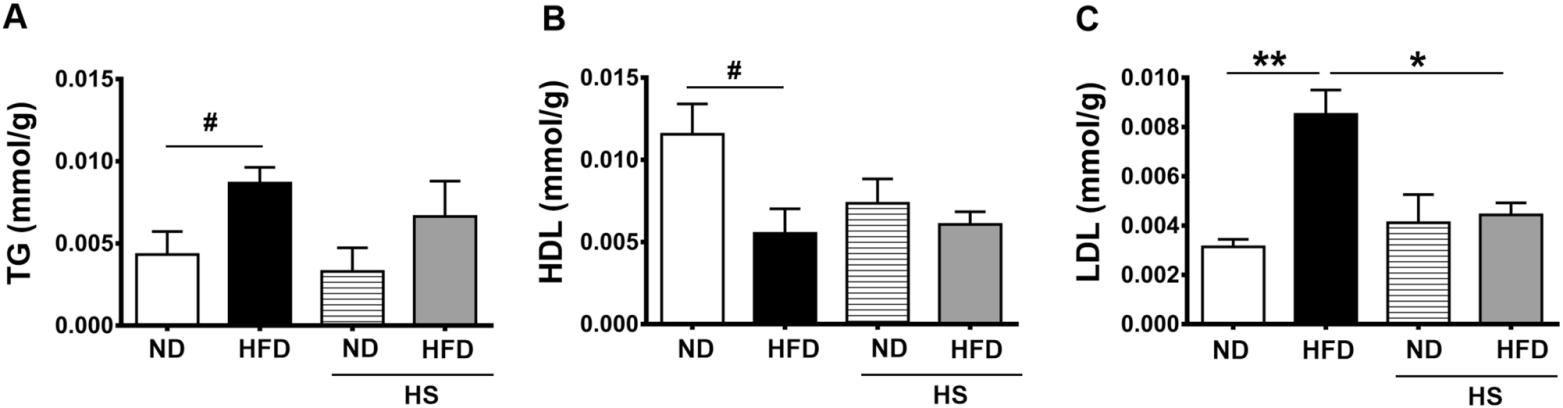
Lipid profile of different diets groups. (**A**) Triglyceride content in liver tissue. (**B**) high density lipoprotein cholesterol content. (**C**) Low-density lipoprotein cholesterol content. ND (n = 5), HFD (n = 5), HS-ND (n = 5), HS-HFD (n = 5). *, ^#^: *P* < 0.05, **: *P* < 0.01. Panel (**A**,**B**) were measured by Student’s *t* test followed by unpaired *t* test. Panel (**C**) was measured by one-way ANOVA followed by Tukey’s multiple comparison test.

It was observed that lipid profile of HFD group were greatly impacted by HFD. In addition, the TC value was significantly increased in HFD group compared with ND (*P* < 0.05), and HS-HFD groups (*P* > 0.05, Fig. 2A). HDL level of HFD group were significantly lower than ND group (*P* < 0.05, Fig. 2B). LDL level were significantly higher in HFD group than ND group, and also higher than HS-HFD group (*P* < 0.01, *P* < 0.05, Fig. 2C). It is notable that there was no significant difference between HS-ND and HS-HFD group mice TG, HDL, LDL level in liver.

### Mouse jejunum microbiota analysis

**Figure 3:**
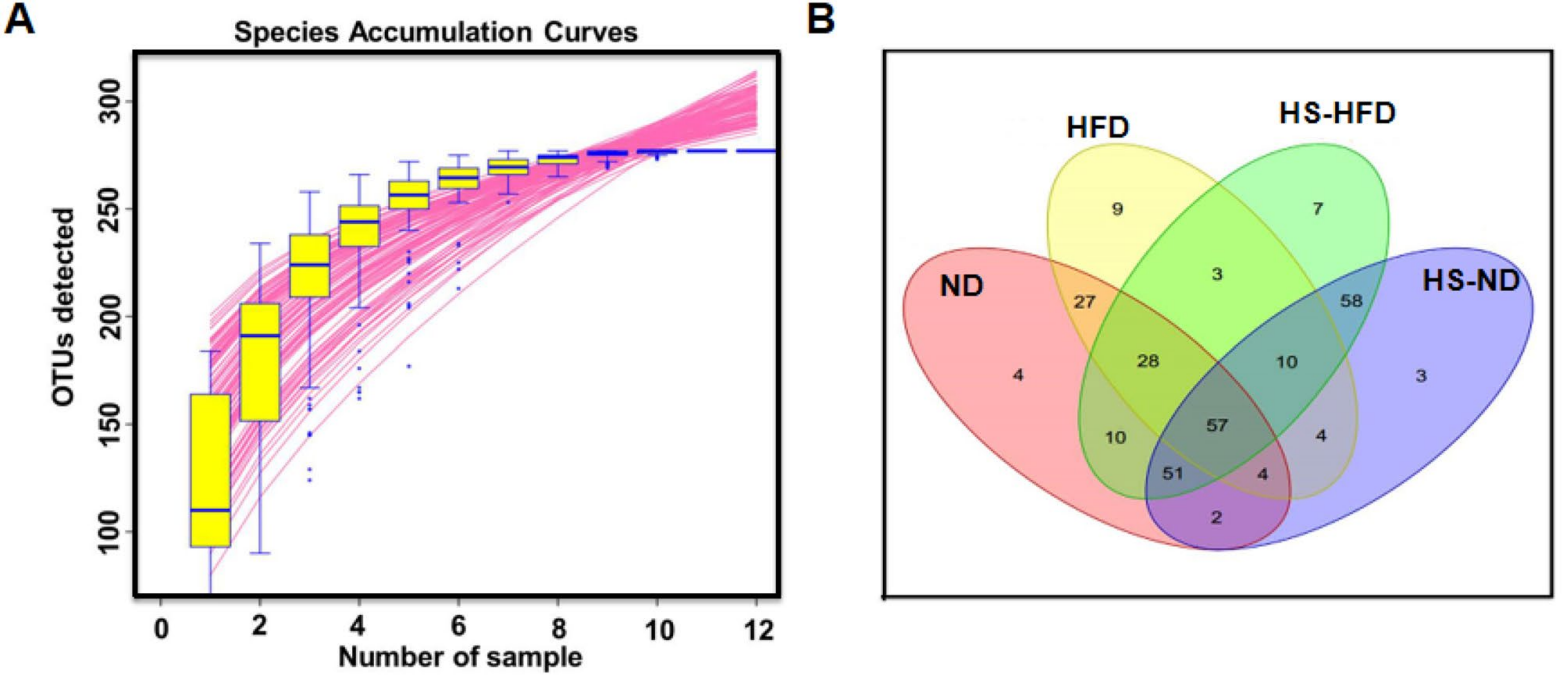
Analysis of 16S rRNA gene sequence raw reads from 12 jejunum samples. (**A**) Species accumulation curves. (**B**) Venn Diagrams based on the shared and unique OTUs across the groups.

The diversity, richness, and composition of jejunum microorganisms were analyzed in normal and obesity-T2DM mice, respectively. Total of 501,322 effective 16S rRNA raw reads were obtained from mice jejunum microflora samples. Effective tags, optimized sequence, other parameters were shown in Table 1. Obtained results were further confirmed with species accumulation curves analysis (Fig. 3A), and Venn diagrams showed the interrelationship of OTUs in jejunum samples among the different groups (Fig. 3B). Analysis indicated that the sequencing and analyzing methodologies in this study were appropriate and acceptable.

**Table 1:** Tags and alpha diversity index information.

	ND	HFD	HS-ND	HS-HFD
Effective tags	9209 ± 3241	11,118 ± 5735	27,187 ± 8857	19,985 ± 10,266
Majorized sequence	39,628 ± 5656^**#**^	34,700 ± 1455	48,698 ± 942.1	44,082 ± 8249
OTUs number	102.3 ± 8.622	82 ± 40.73	167.7 ± 17.62	120.7 ± 52.7
Coverage rate (%)	100%	100%	100%	100%
Ace	124.8 ± 12.74^**#**^	125 ± 41.83	182.9 ± 18.11	134 ± 43.60
Chao	123.4 ± 19.64^**#**^	118.6 ± 59.22	184.8 ± 16.75	136.9 ± 53.23
Shannon	2.318 ± 0.1167	2.449 ± 1.181	2.407 ± 0.1320	1.774 ± 0.8758
Simpson	0.2339 ± 0.03374	0.2236 ± 0.2453	0.1845 ± 0.02736	0.3470 ± 0.2752
PD-whole tree	37.71 ± 17.94^**#**^	18.05 ± 14.14	71.38 ± 12.74	60.61 ± 26.78
Sobs	102.3 ± 8.622^**##**^	82 ± 40.73	167.7 ± 17.62	120.7 ± 52.70

Values were expressed as mean ± SD or as percentages. ^#^*P* < 0.05, ^##^*P* < 0.01 compared to HS-ND group. All statistics results were measured by Student’s *t* test followed by unpaired *t* test.

**Figure 4:**
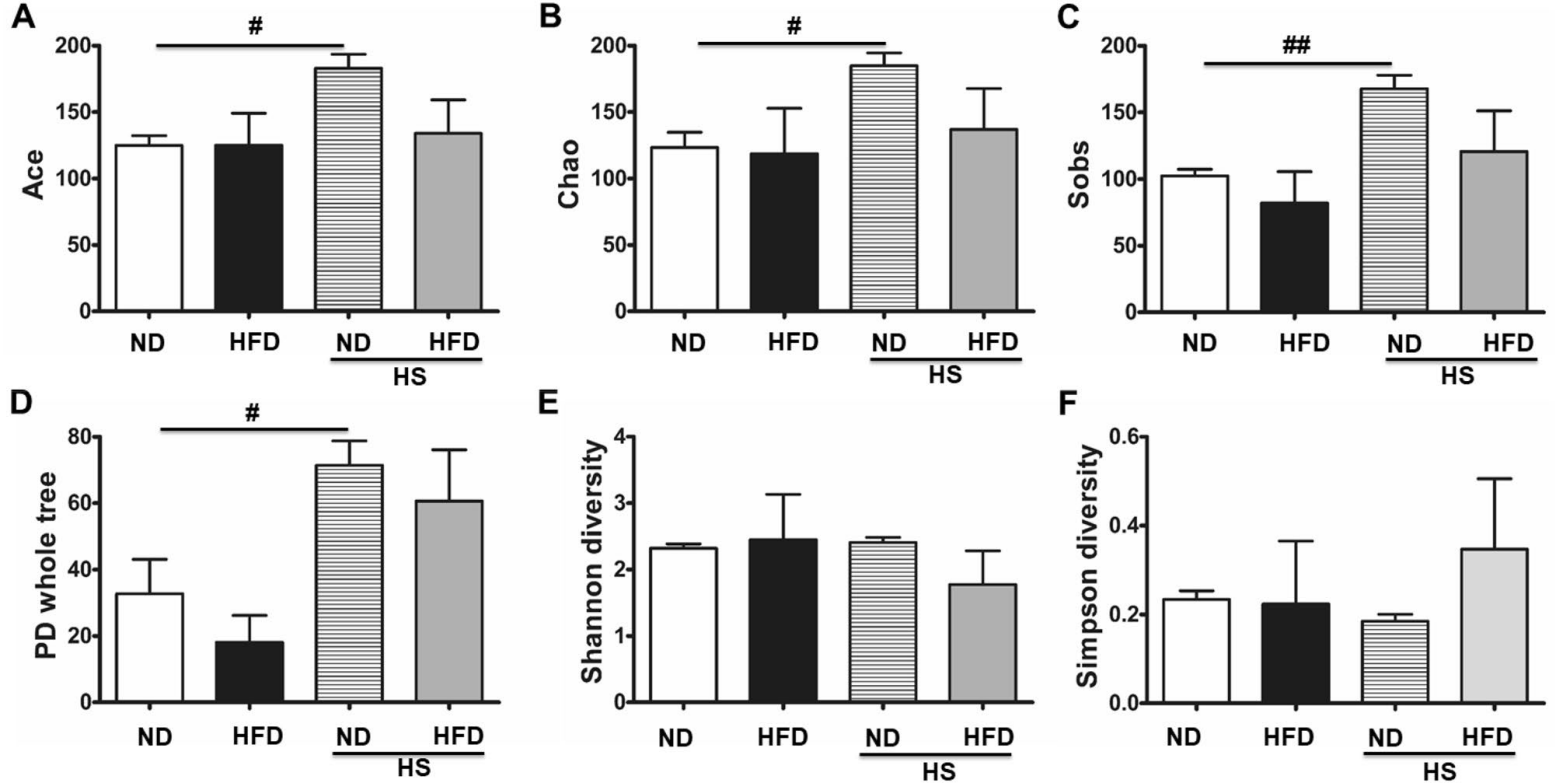
Alpha diversity of jejunum microbiota in different dietary groups. (**A**) Ace index. (**B**) Chao index. (**C**) Sobs. (**D**) PD whole tree. (**E**) Shannon diversity. (**F**) Simpson diversity. Ace and Chao index reflected the richness of the communities in the samples (species richness). Sobs, the actual observed OTU number was equivalent to the number of OTU. Shannon and Simpson index reflected the diversity of communities (species diversity). PD-whole-tree was Faith’s phylogenetic diversity, reflects the difference in the preservation of the evolutionary history of the species in the sample. n = 3/group, ^#^*P* < 0.05, ^##^*P* < 0.01. All statistics results were measured by Student’s *t* test followed by unpaired *t* test.

Total of 277 OTUs in all samples were observed. Among them, 57 OTUs were common for all four groups, and some characteristic OTUs were obtained, such as 4 OTUs were unique for ND group, 9 were found in HFD group only, 7 were special in HS-ND group, and 3 were particular in HS-HFD group, respectively. As shown in Table 1 and Fig. 4, dynamic changes of jejunum bacterial diversity were evaluated for each different group. Alpha diversity was been applied to express richness, diversity, and evenness, respectively. Richness indexes Ace and Chao indicated that total OTU numbers of HS-ND were significantly higher than that of ND group (*P* < 0.05, Fig. 4A,B). Sobs, observable OTUs number, has also been used to illustrate species richness. Results showed that HS-ND group had significantly higher richness than ND group (*P* < 0.01). In addition, PD-whole tree measurement indicated that the diversity level of OTUs in HS-ND was higher than ND group (Fig. 4D). No remarkable difference was observed in Shannon and Simpson diversity analyses (Fig. 4E,F). Above results illustrated that high salt intake increased richness of the microbiomes.

**Figure 5:**
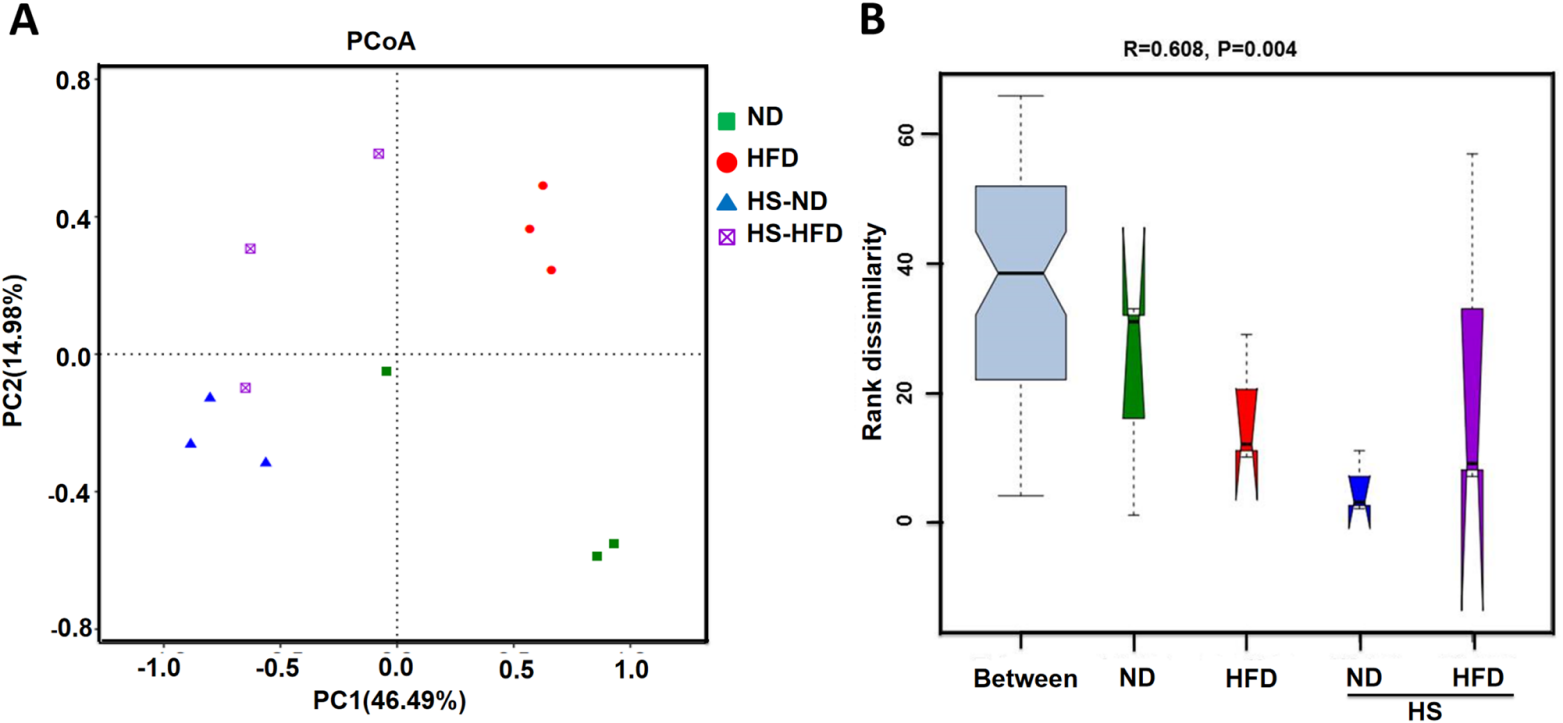
(**A**) PCoA based on Bray–Curtis distance. (**B**) Weighted UniFrac ANOSIM. *R* value was ranged from − 1 to 1. *R* > 0, indicating the difference between four groups was greater than the difference within the group; *R* < 0, significant differences between samples within the group.

Principal coordinate analysis (PCoA) was conducted with Bray–Curtis distance normalization method, which have been used to illustrate the distribution of different samples based on the bacterial community structure (Fig. 5A). ANOSIM of Weighted UniFrac distances (Fig. 5B) revealed a significant difference in the bacterial communities among the groups (*R* = 0.608, *P* = 0.004). These results indicated that the diversity of bacterial community within the same group were less obvious than that of different groups, which elucidated that bacterial community structures were variable enough among different groups.

### Microorganism community composition in the mouse small intestines

**Figure 6:**
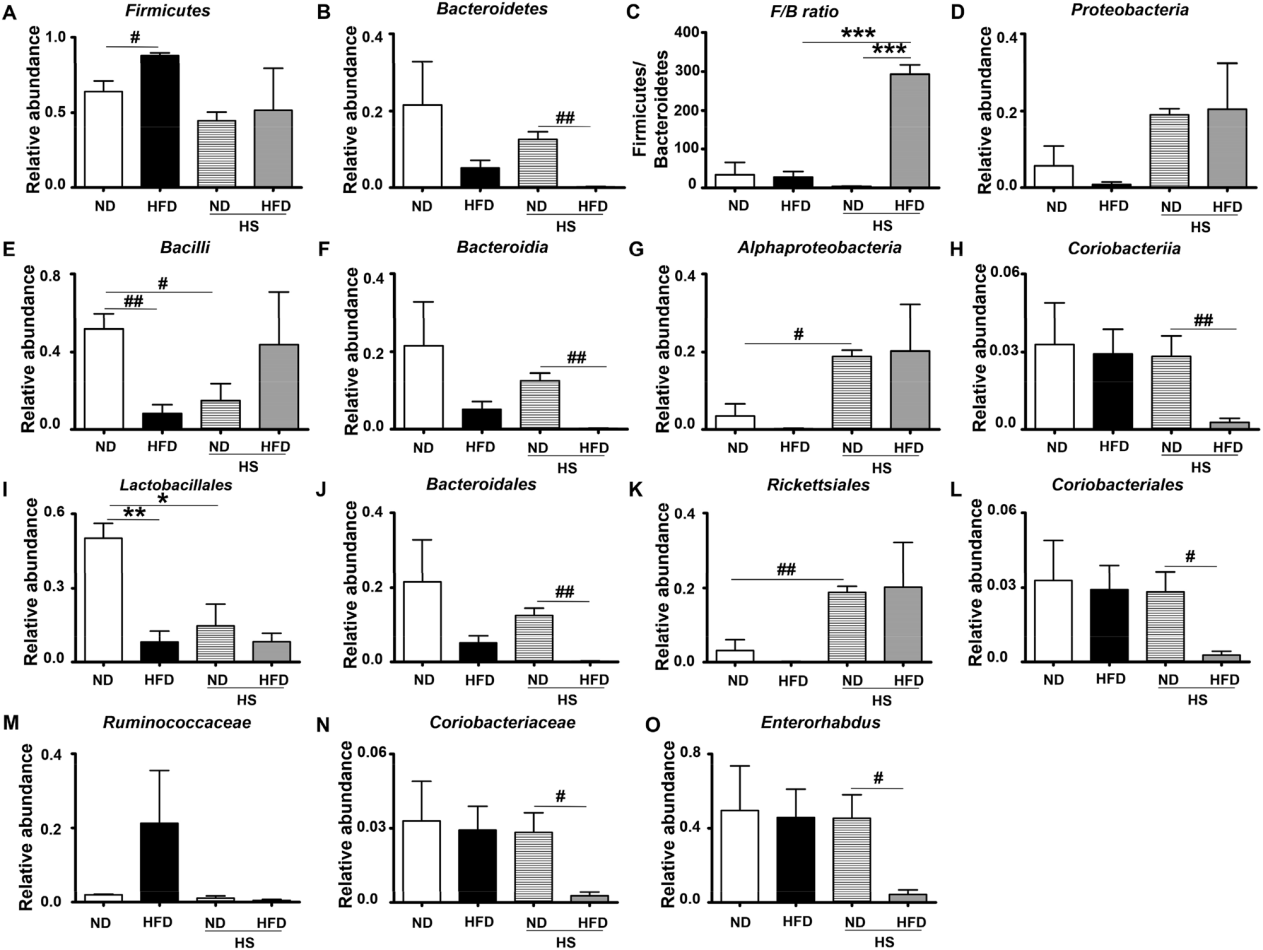
Effects of high dietary salt on microorganisms in mouse jejunum. (**A**–**D**) Phylum level. (**A**) *Firmicutes*, (**B**) *Bacteroidetes*, (**C**) *Firmicutes/Bacteroidetes* (*F*/*B*) ratio, (**D**) *Proteobacteria*. (**E**–**H**) Class level. (**E**) *Bacilli*, (**F**) *Bacteroidia*, (**G**) *Alphaproteobacteria*, (**H**) *Coriobacteriia*. (**I**–**L**) Order level. (**I**) *Lactobacillales*, (**J**) *Bacteroidales*, (**K**) *Rickettsiales*, (**L**) *Coriobacteriales*. (**M**,**N**) Family level. (**M**) *Ruminococcaceae*, (**N**) *Coriobacteriaceae*. (**O**) Genes level, *Enterorhabdus*. n = 3/group, *^,#^*P* < 0.05, **^,##^*P* < 0.01, ***P < 0.001. (**C**) and (**J**) were measured by One-way ANOVA followed by Tukey's Multiple Comparison Test. Other statistics results were measured by Student’s* t* test followed by unpaired *t* test.

To investigate the effects of high dietary salt on SIM composition of obesity-T2DM mice, this work compared the jejunum microorganisms in different groups. At the phylum level, microorganisms mainly belonged to five phyla, which were *Firmicutes*, *Bacteroidetes*, *Proteobacteria*, *Verrucomicrobia*, and *Actinobacteria*, respectively. *Firmicutes* was the most dominated phylum in all groups with total occupation of 64%, 88%, 45%, and 51.6% in SIM of ND, HFD, HS-ND, and HS-HFD groups, respectively (Fig. 6A). The ratio in HFD was significantly higher than that in ND group (*P* < 0.05). According to Fig. 6B, *Bacteroidetes* was dramatically decreased in both HFD groups (total as 5% and 0.2%, respectively) compared to two ND groups (22% and 13%, respectively) and for HS-HFD vs. HS-ND, *P* < 0.01. The *F/B* ratio increased greatly in HS-HFD group than other groups (*P* < 0.001) (Fig. 6C). It was worth to mention that another known obesity-related major phylum, *Proteobacteria*, increased in both HS-groups, while no obvious changes were reveled in HFD group (Fig. 6D). Figure 6E–O showed the changes in class, order, family, genus levels of bacteria, respectively. The lactic acid producing bacteria, *Bacilli* (Fig. 6E) and *Lactobacillales*, (Fig. 6I) markedly increased in ND than HFD and HS-ND groups, which consist with previous reports (Ferguson et al.^38^). Furthermore, HS treatment increased *Rickettsiales* and *Alphaproteobacteria* significantly between HS-ND and ND groups (Fig. 6G,K), and same trend for HS-HFD vs. HFD was observed as well.

**Figure 7:**
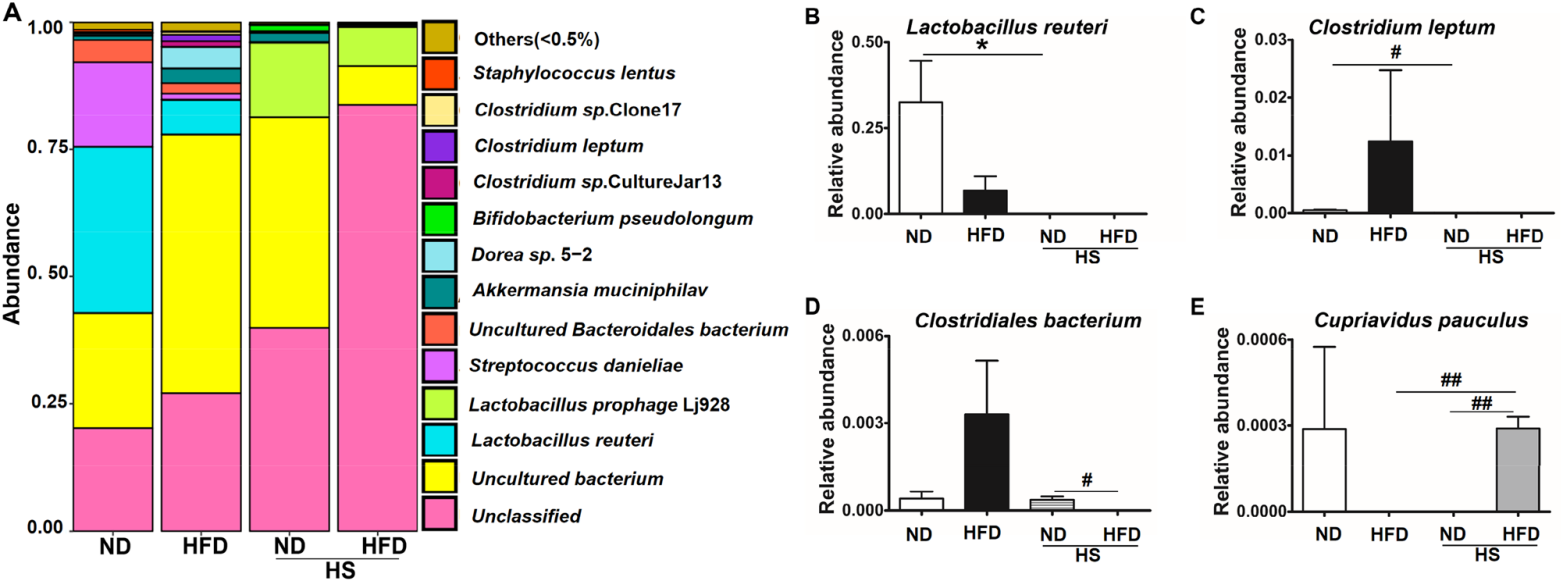
Species level analysis of gut microbiota. (**A**) Species distribution histogram. (**B**–**E**) Relative abundance of 16S rRNA gene for species. n = 3/group, *^,#^*P* < 0.05, ^##^*P* < 0.01. B was measured by one-way ANOVA followed by Tukey’s multiple comparison test. Other statistics results were measured by Student’s *t* test followed by unpaired *t* test.

The relative abundance at species level was assessed as well (Fig. 7A). Among them, two HS groups had relatively lower abundance of *Lactobacillus reuteri* and *Clostridium leptum*, and there was a statistical difference only between HS-ND and ND groups (*P* < 0.05, Fig. 7B,C). *Clostridium leptum*, an obligate anaerobe, appeared significantly less in HS-ND than in ND group (Fig. 7C). In addition, within HS groups, *Clostridiales bacterium* in HS-HFD less than in HS-ND group (*P* < 0.05, Fig. 7D). In the human infection associated bacteria, *Cupriavidus pauculus*, analysis, HS-HFD group showed significantly increased value compared to HFD and HS-ND groups (*P* < 0.01, Fig. 7E).

## Discussion

### Body weight (B.W.), food and water intake

The association between high salt intake with increased risk of obesity and other metabolic syndromes is still a controversial topic in related fields^16^. In the guts, microorganism community has been playing important roles in regulating several metabolic syndromes, such as obesity and diabetes^17,18^. In this work, obesity-T2DM mouse model was constructed to investigate the effect of high dietary salt on the small intestine microbiota to understand more on the relationship among high-salt, SIM and the metabolic diseases.

Analysis indicated that high salt intake could restrict the B.W. Through comprehensive comparing among intake patterns of food, water and energy, it was found that the increasing degree of B.W for HS-ND and HS-HFD were relatively lower than that of ND and HFD groups. Possible reasons for this phenomenon were discussed based on the results of published works. Some previous studies pointed that HFD or HFD-HS could increase the B.W. of animal^19–21^. Meanwhile, several research results also revealed that high salt diet suppresses B.W. no matter fed with ND or HFD^22^. Several workers have tried to explain the effect of high salt on B.W. from renin-angiotensin system^22^, uncoupling protein 1 (UCP-1) and L-thyroxine mechanisms^23,24^. The affecting reason could also be analyzed from other points. It was preliminary considered that high dietary salt might affect the appetite on different extent in different groups. Moreover, the affect was more remarkable in HS-HFD group. Nevertheless, the reason could be more complicated for different groups and samples^25^.

### Fasting blood glucose (FBG), glucose tolerance (GTT), insulin tolerance (ITT), and serum insulin level

In HS-HFD group, the B.W., food, and energy intake were much lower than HFD group, and however, this group still showed clear T2DM pathological characteristics compared to HS-ND group. It was reported that NaCl could affect glucose absorbing in intestinal lumen, and might improve glucose tolerance^26^. HFD mice presented remarkably severer glucose tolerance than HS-HFD mice, and relatively higher serum insulin level than HS-HFD groups. Previous works also suggested that the obesity contributed much more than high dietary salt for the T2DM development^27^.

### Triglyceride (TG), high density lipoprotein cholesterol (HDL-C), low-density lipoprotein cholesterol (LDL-C) evaluation in liver

It is well known that HFD treatment disturbs serum lipid profile with higher TG, LDL-c and lower HDL-c concentration^28^.TG, HDL, LDL result revealed in the present study similar with many previous reports. However, HS-HFD group has showed higher TG level with no significance compared to HS-ND group, and similar level both HDL, LDL with HS-ND group. previous reports have concluded that TG, LD markedly increased in high fat and high salt SD rat^29^. HDL, LDL result of the present study revealed as relatively different with some previous studies, and further studies on this point will be conducted in our future works.

### Mouse jejunum microbiota analysis

In this work, the density of the bacteria in jejunum was 1.96 × 10^5^ ± 1.3 × 10^5^ to 2.53 × 10^6^ ± 1.46 × 10^5^ copies/μg·extracted DNA. The bacterial communities in different regions of human intestine exist in various types, and they are crucial for human health^30^. Contrast to the fecal microorganisms represented large intestine microbiota, studies were inadequate for small intestine symbionts due to the comparably lower accessibility, lower abundance, and anaerobic environment^31^. Regardless of the large surface area of its mucosa, it was believed that the SIM had lower-density compared to large intestine microbiota. This was mainly for the existence of bactericidal substances, such as bile acids, and the rapid luminal flow rate^30^. It was reported that the density of the bacteria in the region of SI was about 10^3^ in duodenum, 10^4^–10^5^ in jejunum, and 10^7^–10^8^ in ileum^18^.

### Microorganism community composition in the mouse small intestines

The effect of SIM on animal health still remains to be explored. Moreover, the effect of HS on the SIM in individuals suffering T2DM is also unclear^32^. Combined with earlier studies^33,34^, it was assumed that HS intake could increase the richness yet decrease the diversity of SIM. In addition, HS intake might cause the microbiota compositions switched into an unhealthy direction.

The increased value of *F/B* ratio, which was mainly from the two most predominant phyla, *Firmicutes* and *Bacteroidetes*, in healthy individuals^34,35^, has been considered as a risk factor for obesity, hypertension, and metabolic disorders^36,37^. In this study, HS consumption decreased both *Firmicutes* and *Bacteroidetes*, while HFD increased *Firmicutes* and decreased *Bacteroidetes* as in large intestines. It was worth to mention that HS-HFD reduced *Bacteroidetes* in jejunum dramatically, and this change caused a significant increase of *F/B* ratio (*P* < 0.001). Results indicated a trend that high dietary salt could decrease *Firmicutes* in SIM, which was opposite to the change of *Firmicutes* in large intestines^37,38^. *Proteobacteria*, other diseases related bacteria, was elevated by HS in the jejunum, which been identified as a possible marker for predisposing to disease onset^39^, such as autism, IBD, CVDs, and T2DM. Results revealed that in obesity-T2DM mice, unhealthy microbiota composition had been aggravated by high dietary salt.

Those bacteria, which were affected or changed significantly in HS-HFD group (*Bacteroidia*, *Coriobacteriia*, *Alphaproteobacteria*, *Coriobacteriales*, *Coriobacteriaceae*, & *Enterorhabdus*), were all known to be crucial bacteria for fat-intestinal microbiota-diseases. It was noteworthy that compared with the HFD and ND groups, the HS treatment aggravated the deterioration caused by HFD in small intestines. To the best of our knowledge, this result was reported for the first time. *Rickettsiales* belongs to *Alphaproteobacteria* class, and *Alphaproteobacteria* belongs to phylum *Proteobacteria*. It was assumed that these subgroups contributed more on the changes of the *Proteobacteria* phylum by HS treatment^40^. Further studies were expected to explore the changing types and mechanisms on *Proteobacteria* phylum of these subgroups.

It was observed that HS intake led to decrease of *Lactobacillus* species in intestines, which was consistent with published work^41^. Published works suggested that probiotics have beneficial effects on disorders including reduced insulin resistance, hepatic steatosis^42^, and prevention of obesity^43^. Non-pathogenic *Clostridia* could construct an anti-inflammatory environment by regulating T cells’ activity and secreting fatty acid. Onal et al. reported that HS-HFD significantly decreased the *Clostridia* bacteria strains, and, relative reduction of *Clostridium leptum* related with inflammatory bowel disease^36^. In this study, HS decreased *C. leptum* significantly in jejunum, which might induce a derangement on anti-inflammatory system of SIM.

In obesity-T2DM mice, HS significantly increased the Gram-negative bacterium, depleted the *Bacteroidetes*, elevated *F/B* ratio, reduced the beneficial gut bacteria (such as *Lactobacillus sp.*, *Clostridium sp.*, and increased the pathogenic bacteria *Rickettsiales*). Jejunum is an important segment of the small intestines. For dwelling of micro-symbionts, jejunum contains relatively more bacteria than duodenum. It supports the bacterial colonization, decides the bacterial diversity and density, especially the growth of health-related Gram-positive aerobics and facultative anaerobics like *Lactobacilli*^22,32^. Considering the lower abundance of microorganisms in small intestine, HS intake could induce a more rapid and profound imbalance in SIM in an unhealthy animal than that in healthy ones. However, the effect and mechanisms of SIM imbalance on the bacterial colonization, dynamics, richness and diversity in large intestine are still a challenging topic in related research fields.

Effect of HS treatment on archaea and halophilic microorganisms in jejunum were also investigated. The results showed that there were *Euryarchaeota* and *Thaumarchaeota* phyla, and a species *Halorubrum luteum* found in SIM. However, no obvious differences were observed among groups. *Euryarchaeota* was considered the predominate archaea in intestine and widely distributed in colon^44^. *Halorubrum luteum* was a halophilic archaeon belong to *Halorubrum* genus^45^. Currently, some new species were detected in fecal and colonic contents, and classified to be a member of *Halorubrum* genus^46^. To our best of knowledge, *Halorubrum luteum* were observed in small intestine for the first time.

In summary, this work was conducted to study the behavior and changes of microbiota in the small intestine affected by HS-HFD in obesity-T2DM animals. The results were concluded as follows. Firstly, the B.W could be restricted by HS, and this trend was more obvious in HS-HFD group. Secondly, significant T2DM pathological characteristics were observed in HS-HFD group, regardless of relatively lower food and energy intake, and this probably from the influence of NaCI on glucose absorbing. Thirdly, jujunum microbiota analysis indicated that SIM had relatively lower density compared with large intestine microbiota. Finally, microorganism community composition study suggested that HS intake could increase the richness and yet decrease the diversity of SIM. Moreover, high dietary salt could aggravate the SIM composition imbalance, and further lead to an unhealthy direction. Additionally, some limitations still existed in this study, such as the sample size was relatively small, and the experiments needed to be designed more comprehensively. Future studies will focus on deeper researches related to the connections between high salt diet and obesity, and more other related disorders from the prospective of microbiota alteration in the small intestine.

## Materials and methods

### Animal ethics

All experimental animal procedures were approved by the Ethics Committee for Animal Research, Shenzhen Institutes of Advanced Technology, Chinese Academy of Sciences (protocol number: SIAT-IRB-170401-YGS-RPG-A0312-01). All experimental methods were carried out in accordance with relevant guidelines and regulations. All methods are reported in accordance with ARRIVE guidelines.

### Experimental protocol

In this study, 6-weeks old specific pathogen-free (SPF) level 40 male C57BL/6 J mice were obtained from Hunan SJA Laboratory Animal Co., Ltd. Mice were acclimated to housing conditions (22 °C, 55% humidity, 12 h: 12 h light: dark cycle) in SPF level animal center, Peking University Shenzhen Graduate School with ad libitum access to water and diets for 1 week before formal study. After the adaptation period, the mice were divided randomly into 4 diet groups: Fed normal diet (ND, n = 10), Fed high fat diet (HFD, contains 45% fat; D12451; Research Diets, New Brunswick, New Jersey, n = 10), Fed normal diet and high salt drinking (HS-ND, 2% NaCl, n = 10) and Fed high fat diet and high salt drinking (HS-HFD, 45% fat, 2% NaCl, n = 10) groups^47^, respectively (Supplementary Table S1) for 8 weeks^4^. The filtered to sterile drinking water with or without salt was supplemented with 0.01% aspartame (Nantong Changhai Food Additive Co. LTD, China) to give a better palatability. Each group were treated for 8 weeks. B.W., food intake and water intake were recorded weekly; energy intake was calculated approximately according to the net amount of food intake and the physiological fuel values of the ingredients.

### FBG, GTT, and ITT

FBG level, GTT, and ITT were applied to evaluate the murine obesity-T2DM model. Mice tail snip blood samples were used for FBG measuring (blood glucose strips, and Accu-Check glucometer, Roche, Switzerland). The animals were undergone fasting for 5 h with free water before the FBG test. For GTT, the mice were pre-fasted for 16 h, and then glucose solution (2 g/kg·B.W.) was injected intraperitoneally (*i.p.*). Blood glucose was measured at the following different time points, 0, 15, 30, 60, 90, and 120 min (min), respectively. The ITT was performed after 4 h fasting, *i.p.* injected insulin (0.75 U/kg, Humulin, Lilly, Country), and the blood glucose measured at the indicated time points as in GTT.

### Serum insulin level

After 8 weeks feeding, the mice were anesthetized by isoflurane (RWD Life Science, China) before euthanizing. Blood samples were collected by enucleation of eyeball method from mice under anesthesia, kept at room temperature for 2 h, centrifuged (1500 rpm at 4 °C) for 30 min to separate serum, and serum insulin concentrations determined with Rat/Mouse Insulin 96 Well Plate Assay (MERCK, Germany, Catalog number: RAB0817).

### Liver TG, HDL, LDL level

The liver tissue was weighed accurately, and 9 times the volume of homogenization medium was added according to the ratio of weight (g): volume (mL) = 1:9. The homogenate was mechanically homogenized under ice water bath conditions, centrifuged at 2500 RPM for 10 min, and the supernatant was taken for measurement. Liver TG, HDL, LDL level determined with Triglyceride (TG) test kit (Nanjing Jiancheng, China, Catalog number: A112-1-1), high density lipoprotein cholesterol (HDL-C) assay kit (Nanjing Jiancheng, China, Catalog number: A110-1-1), Low-density lipoprotein cholesterol (LDL-C) assay kit (Nanjing Jiancheng, China, Catalog number: A113-1-1) according to the instruction.

### SIM sampling for metagenome analyzing

The jejunum tissues were collected sterilely^9^. In short, started from pylorus of stomach, proximal 4 cm length of bowl is duodenum. After discard 2 cm length between duodenum and jejunum, the following 6 cm jejunum were collected, snap frozen in liquid nitrogen, and then transferred into − 80 °C until analysis.

For metagenome analysis, the jejunum with its contents was sectioned into 1 cm length under sterile condition, put each section of jejunum into individual sterile Eppendorf tubes, kept in dry ice, and then sent for high-throughput sequencing analysis (3 samples for each group).

### High-throughput sequencing

Microbiota analysis was performed by Tiny Gene Bio-Tech Co., Ltd. (Shanghai, China). The genomic DNA of the jejunum bacteria was extracted. Specific primers (see Supplementary Table S2) were used to amplify the V3-V4 region of bacterial and archaea 16S rRNA gene, and then each target fragment was recovered as template for secondary round PCR amplification. After that, PCR products were recovered (AxyPrep DNA gel recovery kit, AXYGEN, USA, CB55766755), and quantified with a FTC-3000™ real-time PCR instrument (Funglyn Maple ridge, Canada). The samples were mixed according to the equal mole ratio to complete library preparation before sequencing.

The 2 × 300 bp based library was high-throughput sequenced (Illumina Miseq, Illumina, California) and carried out bioinformatics analyses. The effective sequences obtained were controlled by Trimmomatic software (v.0.35), and sequences splicing by FLASH software (v.1.2.11, Adobe, USA). MOTHUR software v.1.33.3 was used for the quality of reads after merge. Using UPARSE (usearch, version v8.1.1756), the representative sequences of full name Operational Taxonomic Units (OTU) were obtained by clustering with 97% similarity. The obtained OTU representative sequences were then compared with the progressive object of the database by MOTHUR (classify. seqs) for species annotation; OTUs without annotation were filtered out. Finally, the OTUs were clustered based on sequence similarity, taxonomic analysis, alpha diversity, beta diversity and inter-group difference analysis.

### Statistical analysis

All data were presented as the mean ± standard deviation. All in vivo experimental data statistical differences among four groups and microbiota results were analyzed with one-way analysis of variance (ANOVA) followed by Tukey’s multiple comparison test, or Student’s *t*-test. The *P* values less than 0.05 were considered significant statistically. Marked as *P < 0.05, **P < 0.01, ***P < 0.001 for ANOVA, and ^#^*P* < 0.05, ^##^*P* < 0.01 for *t* test, respectively.

## Supplementary Information

Supplementary Tables.

## Data Availability

The datasets presented in this study can be found in NCBI with the accession number PRJNA864077.
