# Publisher Correction: Small intestinal microbiota composition altered in obesity-T2DM mice with high salt fed

**DOI:** 10.1038/s41598-023-37366-9

**Published:** 2023-06-22

**Authors:** Goher Kerem, Xiangfang Yu, Aynur Ismayi, Bin Teng, Anjaneyulu Udduttula, Chang Liu, Zhongjia Yu, Dilbar Tohty, Jian V. Zhang, Pei-Gen Ren

**Affiliations:** 1 Xinjiang Key Laboratory of Special Species Conservation and Regulatory Biology, College of Life Science, Xinjiang Normal University, Urumqi, China; 2 Center for Energy Metabolism and Reproduction, Shenzhen Institutes of Advanced Technology, Chinese Academy of Sciences, Shenzhen, China; 3 Shenzhen College of Advanced Technology, University of Chinese Academy of Sciences, Shenzhen, China; 4 Guangdong Key Laboratory of Nanomedicine, Shenzhen, China

Correction to: *Scientific Reports* https://doi.org/10.1038/s41598-023-33909-2, published online 22 May 2023

In the original version of this Article Goher Kerem and Xiangfang Yu were omitted as equally contributing authors.

The original Article has been corrected.

