# Supplementary material for: Small intestinal microbiota composition altered in obesity-T2DM mice with high salt fed
Source: Sci Rep. 2023 May 22;13:8256. doi: 10.1038/s41598-023-33909-2 (PMC10203271; doi:10.1038/s41598-023-33909-2)
Supplement: Supplementary file 1 — Supplementary Tables. [file 41598_2023_33909_MOESM1_ESM.pdf]

Supplementary information

Table S1.Experiment groups and diets composition

	ND	HFD	HS-ND	HS-HFD
Fat (Kcal %)	10	45	10	45
Protein (Kcal %)	20	20	20	20
Carbohydrate (Kcal %)	70	35	70	35
Total (Kcal %)	100	100	100	100
NaCl (%) in water	0	0	2	2

ND: normal diet. HFD: high fat diet. HS-ND: high salt drinking, normal diet. HS-HFD: High salt drinking, high fat diet

Table S2. Bacteria and archaea16S rRNA primer pair

Target gene		List of specific primer sequence’s
Bacteria	16S V3-V4	357F 5'-ACTCCTACGGRAGGCAGCAG-3’
		806R 5'-GGACTACHVGGGTWTCTAAT-3'
Archaea	16S rRNA	519F:5’-CAGCCGCCGCGGTAA-3’
		Arch915R:5’-GTGCTCCCCCGCCAATTCCT-3’
		Arch1386R:5’ -GCGGTGTGTGCAAGGAGC-3’
